# The Names of the Grip

**Published:** 1891-08

**Authors:** 


### The Names of the Grip.

There is more in a name than Juliet would have us believe, and in medicine especially, we can often learn much concerning the nature and symptoms of diseases that have raged in epidemic form in past ages merely from the names that have been applied to them at different times, and by different peoples. A good example of this is furnished by the strange malady that has been traveling around the globe for the past eighteen months, and which we now call the influenza or grip. The word influenza is Italian, and means nothing more than influence. We find the term first employed in the writings of Huxham, who, in referring to the epidemic of 1743, speaks of a fever " quæ per totam Europam hoc vere sub nomine inuflenza grassata est." We see from this word how widespread was the affection, since of all epidemic diseases this especially was attributed to atmospheric influence,[1] nothing less universal than the atmosphere itself being deemed capable of diffusing so widely the materies morbi.

On the other hand, the word " grip " (French, *la grippe*), which curiously enough was also first used in the epidemic of 1743, bears witness to the suddenness of the attack, for, although there is some uncertainty as to the derivation of this word, it comes most probably from the French *gripper*, meaning to seize or take suddenly. Other names indicating the suddenness of the seizure were " le horion " (the thump), applied to an epidemic in France in the fifteenth century, and " Blitzkatarrh," given by the Germans to another epidemic in 1781.

The predominence of catarrhal symptoms in many of the epidemics is shown by this last mentioned name, and also by the terms catarrhus epidemicus, synocha catarrhalis, febris catarrhalis epidemic, le grand rhume, etc., applied to the disease by many writers in the seventeenth and eighteenth centuries. " Russian catarrh " indicated not only the supposed nature of the affection, but also the region in which it was believed to be

1 This is the most commonly accepted explanation of the term, though some authorities refer it to a supposed influence to the stars, and the older Italian writers describe the disease as " una influenza di freddo."

endemic, and from which the recurring epidemics were sent forth to visit Europe and America. An obstinate cough was a noticeable sequel of the disease in many of the epidemics, hence we find the names "tussis russa," "epidemic cough," "febris thoracica," "quinte," "coqueluche," "Schafhusten," etc.

An evil conscience is one's first accuser, and herein lies the reason why men have always regarded epidemics as the chastisements of an offended deity. And it is only within a short time that we have learned that sins against sanitary laws bring more speedy, and just as certain punishment as do offences in the moral order. The sufferers from influenza were, of course, on the lookout for the sin which had brought the visitation upon them, and they usually found it, their only difficulty being in pitching upon the one sin most deserving of chastisement among the many to which they were given. In one epidemic occurring in Paris, which was called "the blow," it was believed that those only were attacked who had sung an indecent song that had happened to have a great popularity just before the outbreak of the grippe. So the poor sufferers had not only to endure the pains and discomforts of the disease, but also the jibes and reproaches of their dear friends for having been found out in the sin of singing an obscene ditty. History does not state that anyone regarded himself as unjustly punished for an offence he did not commit, and very likely there was none such.

There were many other names than those we have mentioned which were applied to the influenza in one epidemic or another, but most of them had only a local meaning, the significance of which has been lost to us. Among these we find the "dunce's malady," "pumpkin disease," "polite sickness," "Spanish pip," "new delight," "petite poste," "folette," "coquette," "the general," "fashionable fever," "turning disease," "flosse kolen," "Burzelen" and "disease of the bald-heads." In the island of Martinique it was thought that the disease had been brought by the French soldiers, whence it was called "le chapeau quarre." Often people gave up in despair any attempt to find an appropriate name for the affection and called it simply "the epidemic," "the disease," "the disorder" and the like, terms about as definite and descriptive as "it," which was the appellation chosen by a medical writer in a neighboring city in a recent contribution to one of the New York dailies.—*Editorial Medical Record.*

THE ACTION OF COCAINE ON THE CIRCULATION.—As a result of painstaking study in the direction of ascertaining—experimentally—the exact effects of the drug cocaine upon the circulatory system, Dr. Edward T. Reichert (*American Lancet*) arrives at the following important conclusions: 1. Very much depends upon the individual susceptibility in noting the medicinal effects. 2. Repeated small doses first decrease then increase, and finally decrease the pulse-rate. 3. The cardio inhibitory centres are always affected. 4. The arterial pressure is always increased, unless after large doses, when temporary decrease may happen. This increased arterial pressure may continue longer than the period of acceleration of the heart beat. This increase is chiefly due to stimulation of the vaso-motor centers, to some stimulation of the vessel-walls, and to the increased pulse-rate. These and other noted results show, 5, that cocaine is a decided circulatory stimulant.

